# Engineering microbial phenotypes through rewiring of genetic networks

**DOI:** 10.1093/nar/gkx197

**Published:** 2017-03-21

**Authors:** Oliver P.F Windram, Rui T.L. Rodrigues, Sangjin Lee, Matthew Haines, Travis S. Bayer

**Affiliations:** Centre for Synthetic Biology and Innovation and Department of Life Sciences, Imperial College London, London SW7 2AZ, UK

## Abstract

The ability to program cellular behaviour is a major goal of synthetic biology, with applications in health, agriculture and chemicals production. Despite efforts to build ‘orthogonal’ systems, interactions between engineered genetic circuits and the endogenous regulatory network of a host cell can have a significant impact on desired functionality. We have developed a strategy to rewire the endogenous cellular regulatory network of yeast to enhance compatibility with synthetic protein and metabolite production. We found that introducing novel connections in the cellular regulatory network enabled us to increase the production of heterologous proteins and metabolites. This strategy is demonstrated in yeast strains that show significantly enhanced heterologous protein expression and higher titers of terpenoid production. Specifically, we found that the addition of transcriptional regulation between free radical induced signalling and nitrogen regulation provided robust improvement of protein production. Assessment of rewired networks revealed the importance of key topological features such as high betweenness centrality. The generation of rewired transcriptional networks, selection for specific phenotypes, and analysis of resulting library members is a powerful tool for engineering cellular behavior and may enable improved integration of heterologous protein and metabolite pathways.

## INTRODUCTION

The production of proteins and metabolites using engineered microbial strains is an area of significant interest for many industries, including therapeutics, biomass processing, food and beverage, agriculture and materials (1–5). However, overexpression of heterologous proteins and production of non-natural metabolites remains challenging in many cases. Expression of proteins and metabolic pathways results in a highly unnatural cellular state that invokes a number of cellular stress responses, reducing the quality and quantity of desired products (6,7). For example, accumulation of misfolded protein in cellular compartments can induce the unfolded protein response, leading to a reduction in cellular growth rate and protein production (8). Conventional methods for optimizing industrial microbes include varying external factors (such as pH, temperature and culture aeration), focused genetic modifications such as promoter and secretion tag engineering, or random chemical mutagenesis and screening to discover mutant strains with elevated expression and/or metabolite production (9–11). High level expression of heterologous proteins may require the manipulation of multiple cellular processes at once, including metabolism, stress response and protein processing (12). Therefore, strategies to engineer regulatory networks such that they are ‘tailor made’ for heterologous protein and metabolite production are of significant interest. To date, many approaches have focused on fine-tuning the expression of the heterologous protein or pathway, while relatively few have addressed manipulation of the endogenous regulatory and metabolic network that synthetic pathways are embedded in.

**Figure 1.**
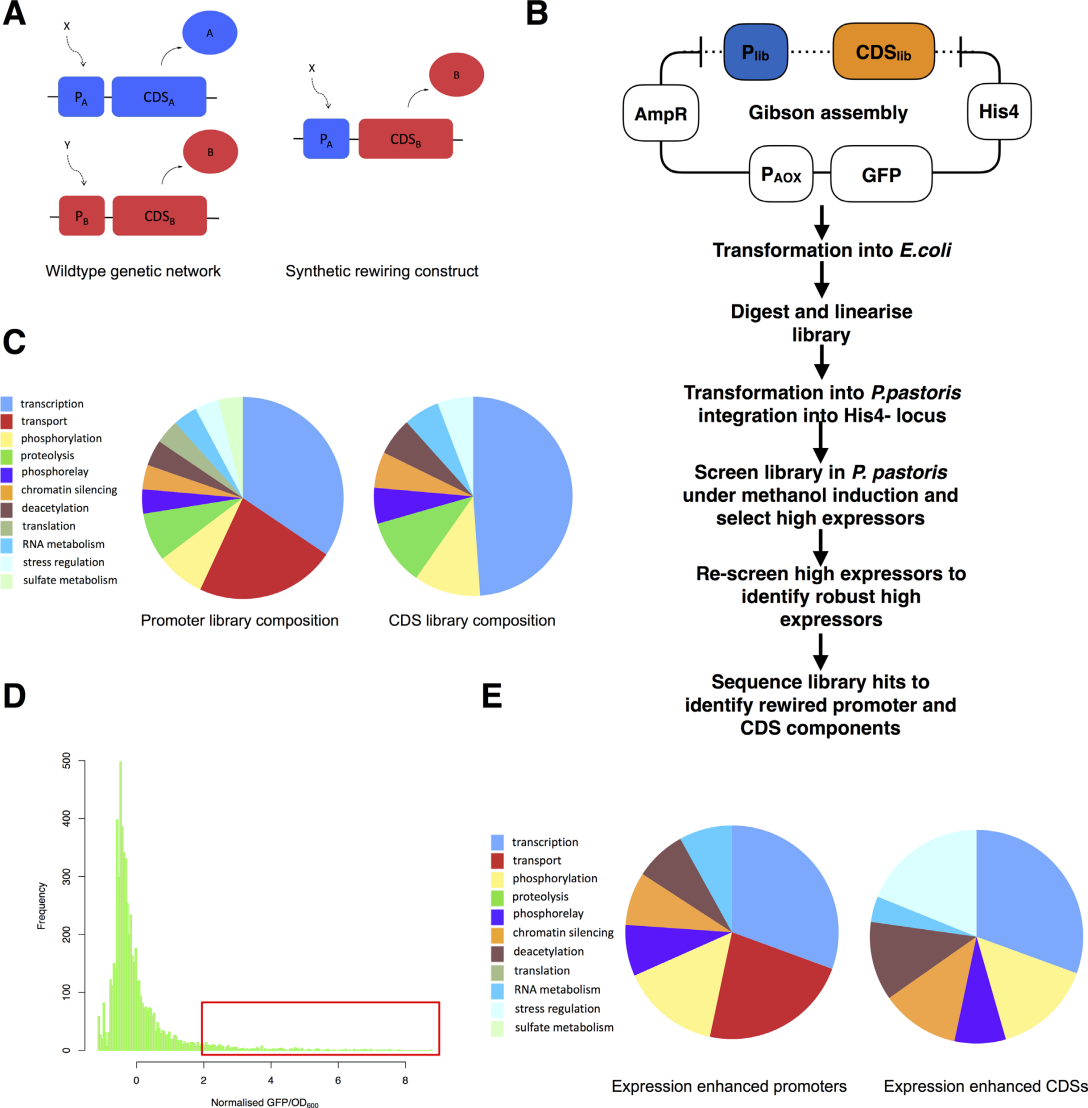
Rewiring cellular regulatory networks using synthetic DNA constructs. (**A**) General strategy for genetic rewiring. Promoter of gene A is fused to CDS of gene B, facilitating transcriptional regulation of gene B by the intracellular signal X. (**B**) Overview of rewiring library screening pipeline. Individual rewired promoter and CDS components are integrated into a single vector with a suitable heterologous reporter construct. Generic 5΄ primer sequences allow random assembly of all possible promoter-CDS pairs via Gibson assembly. The assembled vector library is transformed into *E. coli* to bulk up DNA. The bulked purified vector library is then linearized by restriction digestion and transformed into *P. pastoris* leading to integration of the joint library and reporter vector at the *his4-* locus. Colonies are picked and cultured in 96-well format to induce expression of heterologous reporter. Clones with enhanced heterologous expression are selected and re-screened to verify enhanced expression. Robust clones with enhanced heterologous reporter expression are sequenced to identify promoter and CDS library components. (**C**) Structure and composition of rewiring promoter and CDS library, as described by gene ontology. A full list of promoters and CDSs is given in Dataset S1. (**D**) Growth normalised GFP fluorescence for the rewiring library. Outliers (enclosed red > 2SD) chosen for subsequent re-screening, selection and sequencing to identify rewired expression outliers. GFP fluorescence used as a proxy for protein expression. (**E**) Gene ontology summary for rewiring clones identified as enhanced protein expressors. Compared to the initial library, high expression clones are enriched for promoters and CDSs involved in stress response, protein deacetylation, chromatin silencing and protein phosphorylation and phosphorelay signalling.

Genetic rewiring is a strategy for introducing novel interactions into a transcriptional regulatory network (13). This is accomplished by transforming a strain with a synthetic genetic construct that consists of a promoter fused to a coding sequence (CDS) of a transcriptional regulator (Figure 1A). The synthetic promoter::CDS pair is a non-natural combination of a promoter and CDS found in the strain. This synthetic construct effectively ‘rewires’ the regulatory architecture of the strain creating new routes through which regulatory information can flow (14). Such synthetic network architectures may alter the way in which an organism detects and responds to its environment. Genetic rewiring has been used to examine the robustness of the *Escherichia coli* transcriptional regulatory network to the introduction of new connections. It was found that the network tolerated a large majority of new connections, and that some connections resulted in phenotypes such as enhanced survival in stationary phase (13).

Here, we asked whether genetic rewiring could be used to access novel cellular phenotypes that are industrially valuable, such as the ability to highly overexpress heterologous proteins and to produce non-native metabolites. To address this hypothesis, we created combinatorial libraries of synthetic promoter::CDS constructs in yeast and screened these for enhanced product synthesis. Analysis of library clones with enhanced production phenotypes revealed how genetic rewiring enables protein or metabolite overproduction, and highlights general ‘design strategies’ for the forward engineering of cellular regulatory networks.

## MATERIALS AND METHODS

### Heterologous protein synthesis

Sequences of heterologous proteins for expression in *Pichia pastoris* were synthesized by DNA 2.0 using codon preferences for *P. pastoris*.

### Library construction in *E. coli*

Library components were PCR amplified from *P. pastoris* GS115 genomic DNA using primers OW001 to OW220 detailed in Dataset S2. Primers were designed to amplify 1000 bp of selected promoters or the CDS of a gene. Selected promoters and CDSs are detailed in Dataset S1. Library components were amplified separately. A second PCR was performed to provide an overlapping 5΄ region on promoter sequences for Gibson assembly using primer OW272 (Dataset S2). An assembly PCR was performed to fuse CDS constructs to a downstream AOX1 3΄ terminator fragment amplified using primers OW221 and OW274. The library was built using an 8 h isothermal Gibson assembly integrating library components into the Sma1 site of the modified pAO815 expression vector (Invitrogen) (Figure 1 and Supplementary Figure S1). Proteins chosen for heterologous expression were cloned into the EcoRI site of this vector. The Kozak sequence AAAATGTCT was used and *Saccharomyces cerevisiae* M-α secretion tags where applicable. This provided a single vector that contained both library components and heterologous reporter (Figure 1 and Supplementary Figure S1).

### Yeast stains transformation, growth and maintenance

Transformations were carried out as in (15), with growth and maintenance following Invitrogen protocols. Briefly, the *his4-*strain GS115 used for transformations was maintained on YPD. Electrocompetent cells were re-suspended in 0.01 ml volume of BEDS solution. Competent cells were transformed with ∼100 ng of *SalI* or *StuI* linearized vector (linearized within the His4 marker) in 0.1 cm electroporation cuvettes using the standard fungal settings on a Bio-Rad MicroPulser Electroporator. Cells were recovered in 1 M Sorbitol for 1 h then plated onto RDB agar plates. Colonies from plates were picked and placed into individual wells in 96-well plates containing 200 μl of MGY and grown at 30°C at 700 rpm in a Mictrotron (Infors) for 48 h. Glycerol stocks were then prepared by adding 75 μl of 60% (v/v) glycerol followed by a 30 min incubation at 30°C prior to storage at –80°C. To prepare samples for methanol induction glycerol stocks were replica plated into 200 μl of MD and grown for ∼36 h. Cells were then spun down for 5 min at 1500 g, the supernatant removed and washed once with 150 μl of MM centrifuged a second time and then re-suspended in 200 μl volume of MM. Cultures were subsequently grown for 24 h prior to taking GFP fluorescence and OD_600_ to estimate protein expression and fungal growth.

### Quantifying protein production and identifying outliers

GFP fluorescence (excitation 485 nm; emission 510 nm) and OD_600_ were read in optically clear-bottomed 96-well plates (Greiner Bio-One) on a POLARstar Omega (BMG Labtech). For library screening GFP fluorescence was normalised by dividing by OD_600_ for each well. In order to make readings comparable across plates OD_600_ normalized fluorescence signals were standardized by subtracting the mean and dividing by the standard deviation on an individual plate basis. Primary library samples which showed expression greater than two standard deviations from the mean were assayed a second time. In the second repeat assay, all clones showing >1 standard deviation from the mean of the plate were sequenced to identify the relevant promoter and CDS of the rewiring construct. Sequenced constructs were then rebuilt and independently transformed. For these independent rewiring lines 44 clones were compared to 44 clones containing a relevant control lacking the rewiring construct. A paired two-sample *t* test was used to determine the significant difference in GFP fluorescence between control and rewired lines.

### Lycopene production

A modified version of pAO815 without the AOX1 3΄ region was used with replacement of the promoter and terminator regions upon assembly of the pathway. *crtE, crtB* and *crtI* from *Erwinia uredovora* were synthesised and optimized for expression in *P. pastoris* by DNA2.0. A peroxisomal targeting sequence (PTS1) was added in the 3΄ termini of each gene to allow for targeting of the proteins to the peroxisome. All expression cassettes contained the GAP1 promoter and AOX1 terminator. Expression cassettes were amplified using the primers in Dataset S2 and assembled via one-pot isothermal assembly. Here, again a single vector was assembled containing both library components and the terpenoid pathway components. Transformations were performed as described above. Colonies displaying pigment production were picked into individual wells in 96 well plates containing 200 μl of MD and grown at 30°C at 700 rpm in a Mictrotron (Infors) for 48 h. Glycerol stocks were then prepared by addition of 75 μl of 60% (v/v) glycerol followed by a 30 min incubation at 30°C prior to storage at –80°C. Lycopene concentrations were estimated by monitoring absorbance at 484 nm and correcting for growth with absorbance measures at 660 nm read in optically clear-bottomed 96-well plates (Greiner Bio-One) on a POLARstar Omega (BMG Labtech). Primary library screens were conducted in 300 μl and absorbance measures of diluted cultures (1:5) taken after 48 or 72 h of growth in YPD. Flask culture was performed in 250 ml Erlynmeyer flasks with 40 ml culture volumes in YPD and grown for 72 h.

### Gene ontology

A conservative gene ontology mapping on to the *Pichia pastoris* GS115 genome generated using InterProScan downloaded from the Ghent Bioinformatics & Evolutionary Genomics Website (https://bioinformatics.psb.ugent.be/gdb/pichia/). The biological process ontology was used to summarize all annotated genes from the initial library components and library hits.

### Lipase assay

1.1 mM *p*-nitrophenol butyrate in 200 μl sodium phosphate buffer pH 7.5 was pre-warmed to 30°C and added to 50 μl of culture supernatant 72 h post-induction to allow for ample secretion. Chromogenic substrate development was monitored at 405 nm over 20 min.

### Network analysis

Chip-chip interactions used to construct the network were downloaded from the YEASRTAC database (16). The resulting network consisted of 6407 nodes and 48086 edges. *P. pastoris* genes were mapped to this *Saccharomyces cerevisiae* network using Inparanoid to determine their closest orthologs (17). The network was analysed in Gephi (http://gephi.github.io) through which measures for out degree, betweenness centrality, clustering coefficients and eccentricity were derived. Transcription factor hierarchy was estimated using hierarchical levels determined in (18). Significant enrichment was determined by comparing the set of transcription factor orthologs in question to the remaining transcription factors in the genome using a one tailed *t*-test assuming unequal variances.

## RESULTS AND DISCUSSION

### Identification of a rewired strain with enhanced heterologous protein expression

We first focused on the production of heterologous proteins in *Pichia pastoris* (Figure 1B). We constructed a library consisting of promoters and CDSs that regulate cellular functions known to be important in protein overproduction, such as the unfolded protein response, heat shock response, carbon and nitrogen metabolism, and oxidative stress (Figure 1C). A full list of library components is given in the Dataset S1. We amplified 67 promoters and 43 regulatory CDSs from the *Pichia* genome and assembled them into a library consisting of 2881 rewired network members. This library was constructed in tandem with a cassette for high-level expression of a heterologous reporter: green fluorescent protein (GFP) under the control of the methanol inducible promoter AOX1 (Figure 1B). This library-reporter vector was then linearized by restriction digestion (using two separate enzymes to minimize library component loss due to double cutting) and transformed into *P. pastoris* where integration occurs at a known site, the *his4-* locus. Transformation of this expression construct into *P. pastoris* enabled screening of the library using fluorescence as a proxy for protein expression.

The resulting library was screened at ∼2-fold coverage in microtiter plates (Figure 1B). (i) Clones were placed in individual wells in 96-well plates grown in glycerol minimal media and then induced in methanol minimal media. (ii) Following induction GFP fluorescence was measured at 24 h along with OD_600_. (iii) We normalized cell growth to an OD_600_ of 1. We then normalized the fluorescence of each clone by subtracting mean plate fluorescence and dividing by plate standard deviation. This mean centered standardized data allowed us to compare gene expression across plates (Figure 1D). (iv) We selected and analysed clones with enhanced GFP expression (>2 SD from mean) compared to the library background (Figure 1D). These clones were then isolated and re-screened two more times and the final set of consistent outliers were sequenced to identify relevant promoter and CDS library components (see Figure 1E for a summary). This allowed us to select for clones that consistently enhance heterologous expression.

**Figure 2.**
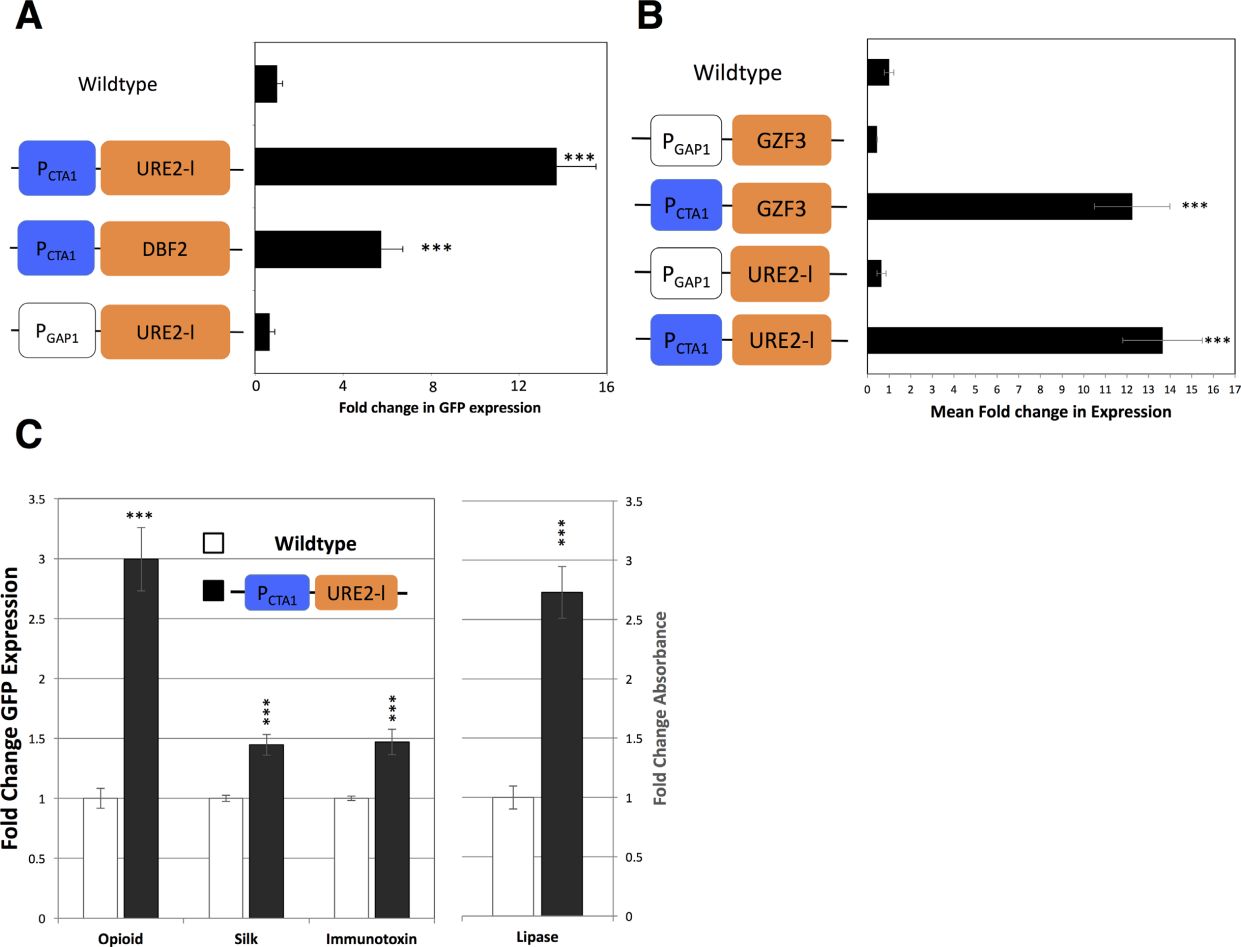
Analysis of library hits. (**A**) Heterologous expression profiles for enhanced rewiring clones, relative to wildtype strain expression. The CTA1::URE2-like strain shows significantly higher GFP fluorescence (∼14-fold over wildtype). When the CTA1 promoter is replaced by the constitutive GAP1 promoter, heterologous expression is similar to wildtype (*N* = 44, ±SE). *t*-test ***P < 0.0001. (**B**) CTA1::GZF3 shows a similar phenotype to CTA1::URE2-like highlighting the importance nitrogen source regulation in heterologous production. Note relevant GAP1 promoter fusions show no altered phenotype. Mean fold change in GFP fluorescence for rewired lines compared to wildtype. (*N* = 44, ±SE). *t*-test ***P < 0.001. (**C**) CTA1::URE2-like rewiring provides a general solution to enhancing production of ‘challenging’ proteins. Mean fold change in GFP fluorescence for C-terminal GFP tagged human membrane bound opioid receptor, spider dragline silk and synthetic immunotoxin proteins along with enzymatic activity of functional lipase. Lipase activity measured by absorbance at 405 nm after 20 min incubation. Mean values are for CTA1::URE2-like compared to control for growth normalized clones. (*N* = 44,±SE). *t*-test ***P < 0.001.

Sequencing the specific promoter and CDS pair from each expression-enhanced clone revealed a variety of ‘solutions’ in the library for increasing GFP expression (Dataset S3). The population of expression enhanced clones was dominated by rewiring solutions involved in oxidative stress and protein metabolism regulation (Figure 1E). To verify robust expression enhancement of selected library isolates we re-cloned the two most frequently isolated rewiring events and compared expression of 44 independent rewired clones with 44 independent control clones containing only the GFP expression construct (Figure 2A). It is noteworthy that these two rewiring solutions were sequenced multiple times in the library screen while all remaining solutions were only identified once (Dataset S3). They thus form a logical focus for further analysis. The most prolific rewiring event isolated was a fusion of a catalase A (CTA1) promoter with URE2-like CDS (CTA1::URE2-l). CTA1 is required for the detoxification of hydrogen peroxide generated during methanol metabolism and is strongly induced upon the shift from glucose or glycerol to methanol (19). In *Saccharomyces cerevisiae* URE2 is a negative regulator of rare nitrogen source metabolism (20). The CTA1::URE2-l construct improved heterologous protein expression by 14-fold over the parent strain (Figure 2A). Another frequent rewiring construct in the set of enhanced expression strains was CTA1::DBF2, which enhanced heterologous protein expression by ∼5-fold. DBF2 is a serine/threonine kinase involved in stress response and regulation of mitosis (21,22). It is possible that spurious mutations contribute to the observed enhanced expression, however, the fact that these rewiring solutions were isolated multiple times in the library screen and our independent re-cloning and testing of CTA1::URE2-l and CTA1::DBF2 (Figure 2A) indicate that it is the rewiring events themselves driving enhanced expression.

The abundance of the CTA1::URE2-l construct in the enhanced expression population suggested that altered regulation of nitrogen metabolism or perception is important in enhancing heterologous protein expression. To test this, we placed URE2-l under constitutive expression using a GAP1 promoter. We found that this strain expressed GFP at levels similar to the wildtype strain, suggesting that dynamic regulation of nitrogen metabolism via the CTA1 promoter is key to achieving higher protein titres (Figure 2B). Furthermore, we found CTA1 promoter fusion to the CDS of a negative regulator of poor nitrogen source use, GZF3 (23), provided a similar enhancement in protein productivity (Figure 2B). This suggests that suppression of enzyme and transport systems required for metabolism of unfavourable nitrogen sources improves heterologous production.

We next asked if the CTA1:: URE2-l strain enables high expression of other heterologous proteins, or whether it is a specific ‘solution’ to enhancing GFP expression. We expressed three different ‘challenging’ heterologous proteins in the CTA1::URE2-l strain, including a synthetic immunotoxin (composed of diphtheria toxin fused to anti-human CD4), membrane bound human mu-opiod receptor, and a spider dragline silk fragment. These proteins have features considered difficult to produce in heterologous expression systems. For example, the spider silk fragment is highly repetitive. Proteins were constructed as fusions with GFP to observe expression level. We observed statistically significant enhancement of expression, with the membrane bound mu-opioid receptor showing ∼3-fold and the immunotoxin and spider silk ∼ 1.5 fold expression over the wildtype strain (Figure 2C). We also found that expression and secretion of a functional heterologous lipase showed enhanced activity by 2.5-fold in the CTA1::URE2-like strain (Figure 2C), suggesting that this rewiring is a general solution to enhancing protein expression. The improved enzymatic activity of the lipase further suggests that this rewiring solution is capable of producing functional protein.

### Screening against different protein targets reveals additional rewiring ‘solutions’

Although the alternative nitrogen regulation represented a specific ‘solution’ to overexpression, we were curious as to whether the rewired strains could reveal general ‘design principles’ for engineering enhanced strains. To do so, we re-screened the library using four additional heterologous reporters. We chose to screen additional libraries using human insulin, human opioid receptor, the synthetic immune toxin and spider silk as heterologous reporters with C-Terminal GFP Tags (Table 1 and Dataset S3). Screens were performed as above with the original GFP library. Several rewiring clones were isolated multiple times within and across multiple libraries (Table 1). This once again supports the idea that these rewiring constructs themselves enhance expression and not other spurious mutations. We independently re-cloned several of these rewiring events and verified reporter expression enhancement by comparing sets of 44 rewired clones with 44 control clones (Table 1). These data suggest that the library screen is in fact identifying rewiring solutions that enhance a wide range of proteins with different properties that might otherwise prove challenging to express. The repetitive isolation of the rewiring solutions CTA1::URE2-l and CTA1::MTR2 in many libraries suggests that these solutions are perhaps well suited for expression of different heterologous proteins with diverse properties (also see Figure 2C). MTR2 regulates mRNA nuclear export, a process that is impeded in *mtr2* mutants at restrictive temperatures leading to progressive inhibition of protein synthesis (24). Also, we noted that several rewiring solutions were isolated multiple times within a specific library, barring specific library biases, which we believe to be minimal considering the common amplicon origin of library components, this suggests that select rewiring solutions could improve expression of proteins with characteristic properties. Two examples include CTA1::GIS2, isolated three times in the silk library (silk being a large highly repetitive protein) vs CTA1::MAC1, isolated three times in the Insulin library (a comparatively smaller simpler structured protein). GIS2, a mRNA regulator has been shown to play a positive role in helping cells respond to stress induced during mitochondrial dysfunction and associates with stress granules in response to glucose deprivation (25,26). MAC1 has a known role in oxidative stress response (27). These solutions may suggest that variable regulation of stress responses can lead to improvements in heterologous production. This independent cloning and validation of expression enhancement also clearly shows that our library screening process identifies rewiring solutions that can enhance heterologous production of proteins with wide ranging physiological properties.

**Table 1.** Analysis of library hits across multiple libraries

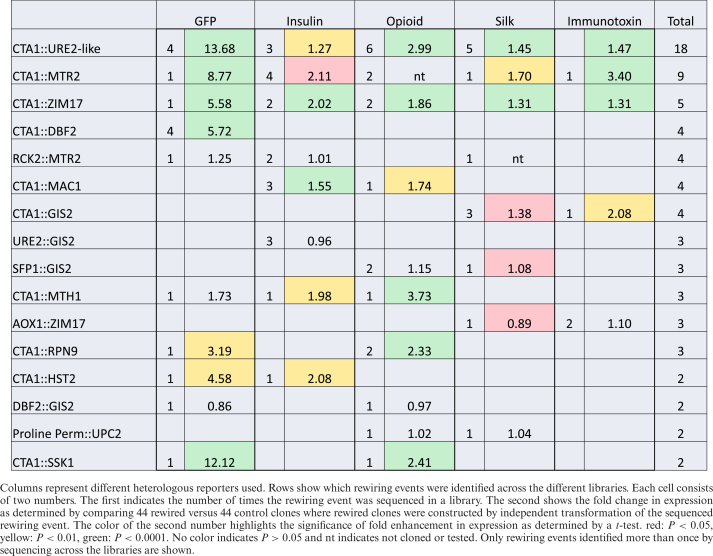

### Screening for enhanced lycopene production shows a diversity of rewiring solutions.

**Figure 3.**
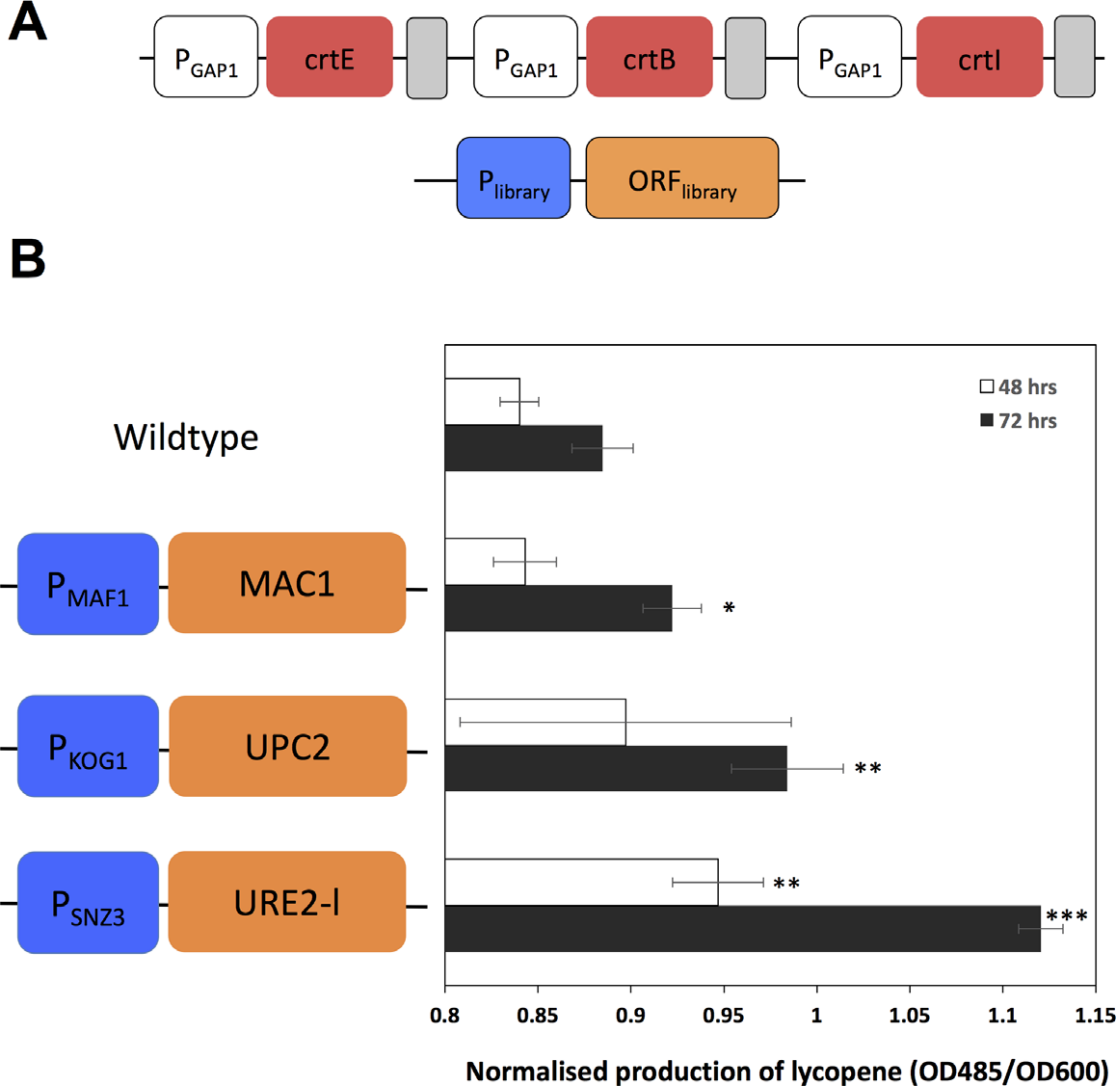
Rewiring terpenoid metabolic pathways for heterologous production of lycopene. (**A**) Genetic constructs in engineered lycopene-producing *P. pastoris* strain. The bacterial genes *crtE, crtB*and *crtI* were placed under the control of constitutive promoters to enable lycopene production. The rewiring promoter and CDS library and lycopene pathway were cloned into the *his4* locus in a single vector. (**B**) Mean production of lycopene in 40 ml cultures. Assayed at 48 and 72 h post inoculation (*N* = 3,±SD). *t*-test: *P < 0.05, **P< 0.01, ***P < 0.0001.

To further validate the rewiring approach and test if it could be used to access industrially relevant phenotypes beyond protein overexpression, we performed a screen of the rewiring library in *P. pastoris* cells engineered to produce the terpenoid lycopene through heterologous expression of *crtE, crtB* and *crtI* from *Erwinia uredovora*. Here again we designed and built a single vector to integrate both the terpenoid pathway and the library into the *his4* locus (Figure 3A). Lycopene provides a convenient screen for metabolite production as pigmentation of cells and cultures can be used to rapidly identify lycopene overproducers. Following 2-fold screening of the library we identified a subset of rewiring constructs that robustly enhance lycopene production (Figure 3B).

The most productive clone SNZ3::URE2-like enhanced lycopene titer by 50% over the parent strain. SNZ3 is a stationary phase induced protein involved in carbon source metabolism (28,29), This solution suggests that altered control of nitrogen regulation may also be important for lycopene production. URE2 also possesses glutathione-S-transferase activity which contributes to overcoming heavy metal and oxidant toxicity (30). Similarly, a second rewiring solution enhancing lycopene production is the copper binding transcription regulator MAC1, which is required for numerous abiotic stress responses including oxidative and heavy metal stress (27). This is driven by the MAF1 promoter, a negative regulator of RNA polymerase III (31). Management of the cellular redox state may be important for robust lycopene production. KOG1::UPC2 also enhanced lycopene production. UPC2 is a positive regulator of sterol biosynthesis (32), which shares metabolic precursors with lycopene production. KOG1 is a component of the TOR1 complex 1 an important mediator of cell growth in response to nutrient signalling, including nitrogen availability, and cell stress (33–37). Overall, these results reveal a diversity of solutions for improving lycopene production and suggest that the rewiring approach can be used to enhance a variety of industrially-relevant phenotypes.

### Network analysis of enhanced strains suggests common topological features among rewired regulators

We next asked whether the rewiring ‘solutions’ described above shared general features in terms of network topology. The identification of related topological network features among rewired regulators could enable more targeted design of rewiring libraries and reveal general ‘design principles’ for high performance strains. Transcriptome networks can be represented as a graph (Supplementary Figure S2) where nodes denote genes and edges between nodes represent transcriptional regulation, with transcription factors binding to and regulating promoters indicated by edge directionality. The topological properties of nodes in networks are known to be important in transcriptional networks as they influence how regulatory information is passed through the network. In particular, regulatory nodes with high connectivity, those that act as communication bridges between distant parts of the network, and nodes at the top of regulatory hierarchies tend to be essential to system function (18,38,39).

**Figure 4.**
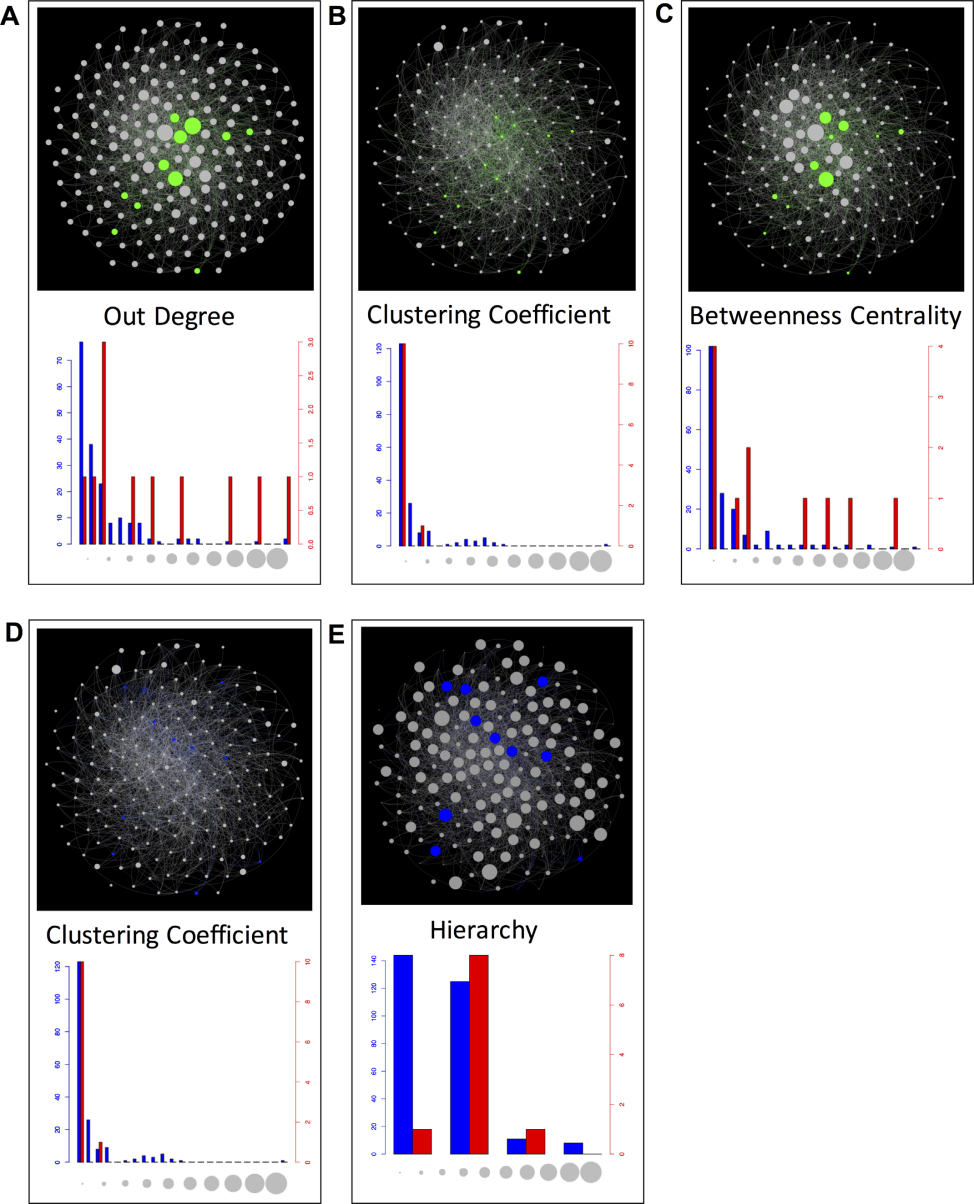
Network analysis and highlighted network topology for groups of different library components. Rewired genes identified in library screens are shown in the context of their endogenous roles—natural and non-modified. Each figure section contains a network diagram (top) and a histogram (bottom). For library CDSs (**A**), (**B**) and (**C**) show out degree, clustering coefficient and betweenness centrality respectively. For promoters (**D**) and (**E**) show clustering coefficient and hierarchy respectively. In the network diagram nodes represent a gene and an edge represents a transcription factor binding to the promoter of a target gene. Colored nodes represent library hits, green for CDSs and blue for promoters. Grey nodes represent the remaining transcription factor comparison set. Only transcription factors are shown in the diagram and compared in the analysis. The size of a node positively correlates to the topological property being quantified. In the histograms blue bars represent the background transcription factor set for the network topology property being measured (gray nodes) and the red bars the library set of interest (green or blue nodes).

Rewired transcription factor library hits from *P. pastoris* for promoters and CDSs identified in all heterologous protein library screens were mapped onto a transcriptome network derived from Chip-chip data (16). For each topological property we compared library hit transcription factors to all remaining transcription factors in the genome. We observed that expression-enhancing CDSs had higher out degree (*P* = 0.016) than the mean out degree of other regulators, suggesting these transcription factors regulate more genes on average. Expression-enhancing CDSs also exhibited higher betweenness centrality (*P* = 0.038) and lower clustering coefficients (*P* = 7.57E–6) (Figure 4A–C, also see Supplementary Figure S2 for explanations of these properties). Betweenness centrality is a measure of how important a node is at linking poorly connected parts of the network. Such nodes function as essential communication bridges between poorly connected parts of the network and can effectively control information flow through the network (39,40). The clustering coefficient is a measure of how likely a node's neighbours are to be connected with one another (41), revealing how a node might facilitate localized information flow between its neighbors.

Analysis of promoters from expression-enhancing clones revealed that they occupy higher positions in the regulatory hierarchy (*P* = 0.026) than the global network mean. Additionally, these promoters also had on average lower clustering coefficients (Figure 4D and E). It is possible that rewiring network components with these properties reduce the distance regulatory information must travel to control network outputs. Previous network analysis in yeast has revealed that adaptive network modules such as those involved in diauxic shift and stress response also exhibit shallow hierarchies of regulators with low clustering coefficients and high out degree (42). This contrasts network modules involved in housekeeping functions such as cell cycle and sporulation, which exhibit opposing topological properties. The fact that the rewiring screen derives solutions that emulate naturally evolved adaptive networks suggests a general strategy for coping with stress in microbial regulatory networks.

## CONCLUSION

We have shown that transcriptional rewiring is a useful tool for engineering physiological traits that underlie protein and metabolite productivity at the cellular systems level. The diversity of rewiring solutions particularly for lycopene production naturally suggests that combinatorial rewiring approaches seeking to combine these solutions with other targeted manipulations will result in additive production enhancements. The dominance of nitrogen regulators in the identified rewiring solutions underscores the importance of optimizing resource allocation for synthetic manufacturing processes. Network analysis of expression enhanced rewired strains suggests ‘design principles’ for engineering microbial strains to cope with heterologous expression stress. In particular, it appears that connecting distant regions of the global regulatory network enables cells to coordinate stress response and adapt to demands imposed by synthetic constructs. Overall, transcriptional rewiring presents a novel approach to uncover industrially valuable phenotypes for use in bioprocessing and bioproduction.

## Supplementary Material

Supplementary Data
