# Supplementary material for: Engineering microbial phenotypes through rewiring of genetic networks
Source: Nucleic Acids Res. 2017 Mar 21;45(8):4984–93. doi: 10.1093/nar/gkx197 (PMC5416768; doi:10.1093/nar/gkx197)
Supplement: Supplementary Data [file gkx197_supp.zip › Supp_Files/Legend Dataset S2.docx]

Dataset S2. Primers used to amplify promoters and open reading frames.
