# Supplementary material for: Engineering microbial phenotypes through rewiring of genetic networks
Source: Nucleic Acids Res. 2017 Mar 21;45(8):4984–93. doi: 10.1093/nar/gkx197 (PMC5416768; doi:10.1093/nar/gkx197)
Supplement: Supplementary Data [file gkx197_supp.zip › Supp_Files/Legend Figure S1.docx]

Figure S1. Modified pAO815 vector. Heterologous reporter under control of AOX1 promoter. AmpR – ampicillin resistance marker, Ori- origin of replication, His4 – histidine biosynthesis auxotrophic selection marker, Lib-Prom promoter library component, Lib-ORF open reading frame library component. AOX1 TT represents AOX1 terminator sequence for both reporter and library ORFs
