# Supplementary material for: Engineering microbial phenotypes through rewiring of genetic networks
Source: Nucleic Acids Res. 2017 Mar 21;45(8):4984–93. doi: 10.1093/nar/gkx197 (PMC5416768; doi:10.1093/nar/gkx197)
Supplement: Supplementary Data [file gkx197_supp.zip › Supp_Files/Legend Figure S2.docx]

Figure S2. Representing the transcriptome in network graphs. Here nodes (blue circles) represent genes and black arrows are edges which represent transcriptional regulation of one gene by another. Nodes 1 and 2 have high connectivity as they interact with large numbers other nodes. Nodes 1 and 2 also regulate a large number of genes and so have high out-degree while 3 has high in-degree. Node 4 functions as a communication bridge between two poorly connected parts of the network, 4 is described as having high betweenness centrality as it lies on communication paths between many other nodes. 5, 1, 3 and 6 form part of a regulatory hierarchy. In Figure 4 *E* the measure of hierarchy ranks nodes by their position in the global transcriptome hierarchy ranking nodes like 5 highly and 6 low. The neighbours (direct interactors of a node) of node 1 are more connected to each other than those of 2. 1 has a higher clustering coefficient than 2, in this respect 2 has ‘more influence’ in governing the local interactions in its neighbourhood.
