# Supplementary figures and images for: Engineering microbial phenotypes through rewiring of genetic networks
Source: Nucleic Acids Res. 2017 Mar 21;45(8):4984–93. doi: 10.1093/nar/gkx197 (PMC5416768; doi:10.1093/nar/gkx197)

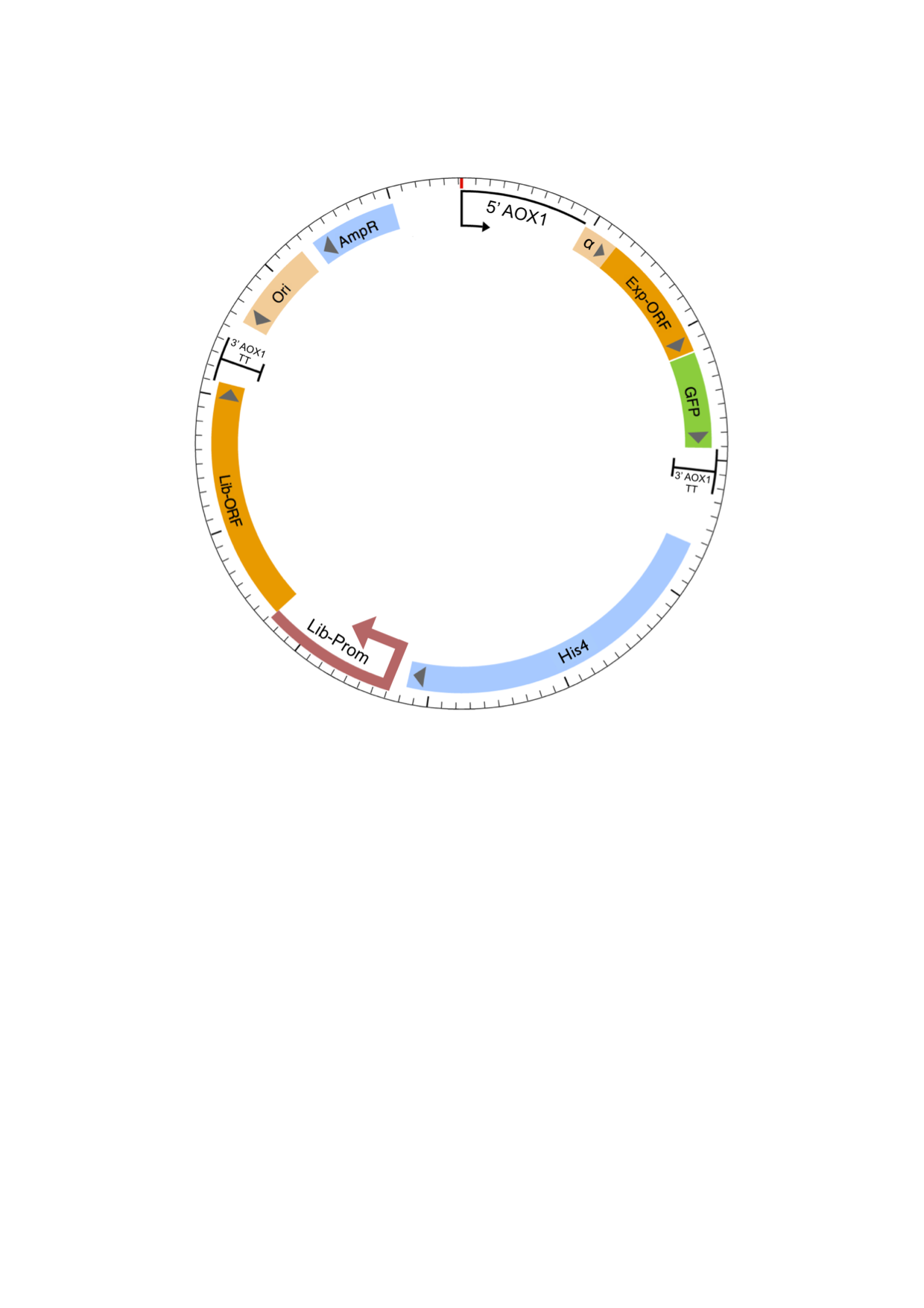

Supplement: Supplementary Data [file gkx197_supp.zip › Supp_Files/Figure_S1.png]

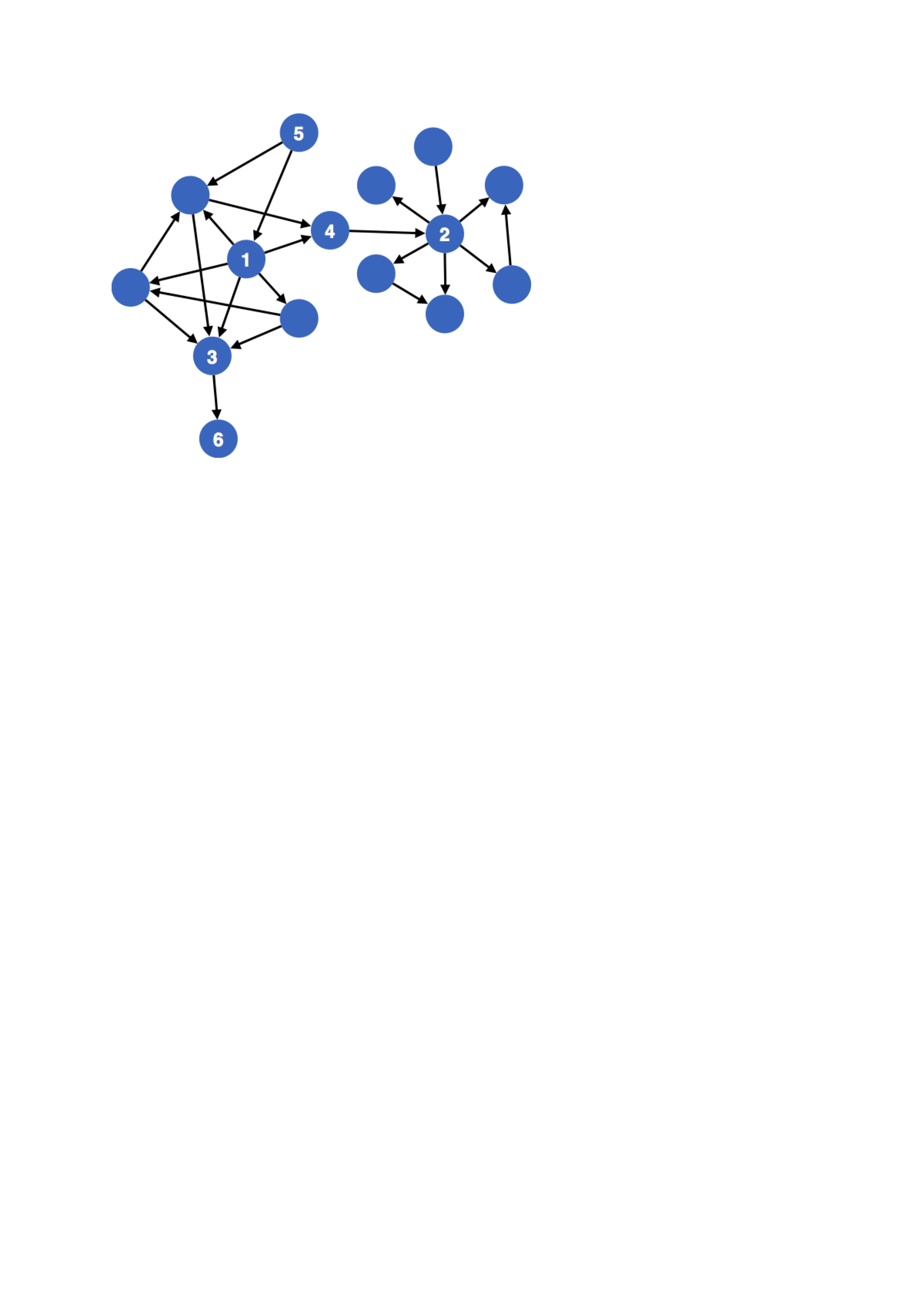

Supplement: Supplementary Data [file gkx197_supp.zip › Supp_Files/Figure_S2.png]
